# Ultrasonic-Assisted Marine Antifouling Strategy on Gel-like Epoxy Primer

**DOI:** 10.3390/molecules29194735

**Published:** 2024-10-07

**Authors:** Zhen Tang, Pengjiao Zu, Baiyi Chen, Xianhui Zhang, Jianfeng Lan, Jiaxun Zhang, Hao Zhang, Baoxin Wang, Li Ma, Jianhua Wu

**Affiliations:** 1 Marine Engineering College, Jimei University, Xiamen 361021, China; 18363870097@163.com (Z.T.);; 2 Fujian Provincial Key Laboratory of Advanced Marine Functional Materials, Jimei University, Xiamen 361021, China; 3 Xiamen Key Laboratory of Marine Corrosion and Smart Protective Materials, Jimei University, Xiamen 361021, China; 4 National Key Laboratory for Marine Corrosion and Protection, Luoyang Ship Material Research Institute (LSMRI), Qingdao 266237, China; 5 College of Materials Science and Chemical Engineering, Harbin Engineering University, Harbin 150001, China; zupengjiao@hrbeu.edu.cn

**Keywords:** ultrasonic treatment, *Pseudoalteromonas*, gel-like marine epoxy primer, adhesion behavior, marine biofouling

## Abstract

Ultrasonic technology has drawn extensive interests for its great potential in marine antifouling applications. However, its effects on the adhesion behavior of marine fouling organisms on marine structures remain underexplored. This work investigated how ultrasonic treatment impacted the adhesion of *Pseudoalteromonas* on a gel-like marine epoxy primer. And the process parameters for ultrasonic treatment were optimized using response surface analysis with Design-Expert software 11. The results revealed that ultrasonic treatment disrupted the cellular structure of *Pseudoalteromonas*, causing the deformation and fragmentation of the cell membrane, leading to bacterial death. Additionally, ultrasonic treatment reduced the particle size and Zeta potential value of *Pseudoalteromonas*, which disrupted the stability of bacterial suspensions. It also increased the relative surface hydrophobicity of *Pseudoalteromonas* cells, resulting in a reduction in adhesion to the gel-like marine epoxy primer. This study demonstrated that ultrasonic treatment significantly disturbed the adhesion behavior of microorganisms like *Pseudoalteromonas* on the gel-like marine epoxy primer, which provided an effective approach for controlling marine biofouling.

## 1. Introduction

Marine biofouling is the unwanted attachment and formation of biological marine growth, where organisms across the major kingdoms of life (viruses, archaea, bacteria, and eukaryotes) form interconnected communities with entwined ecological and interspecies dynamics [1,2,3,4]. Fouling organisms on hull surfaces increase vessel drag, leading to higher energy consumption [5,6]. For instance, medium-sized naval vessels suffering from severe fouling consume 20.4% more fuel than those not fouled [5]. This tremendous fuel consumption contributes to substantial carbon dioxide emissions and other pollutants, exacerbating the formation of particulate matter and acid rain [7]. It is estimated that 4000 species of organisms in the ocean can cause biofouling [8,9], with *Pseudoalteromonas* being a common aerobic microorganism found in various marine environments [10,11]. They are tolerant and can propagate rapidly on unprotected hull surfaces, forming biofilms with extracellular polymeric substances (EPSs). These biofilms provide nutrients for other macrofouling organisms like barnacles and mussels, accelerating their adhesion and growth on hulls [12,13,14]. As a result, vessel performance is impaired, and dry-docking intervals are shortened. Therefore, it is crucial to find effective methods to prevent or inhibit the adhesion of *Pseudoalteromonas* and other microorganisms to hull surfaces, addressing the issue of marine biofouling.

At present, there are mainly two kinds of antifouling coatings used to prevent and control marine biological pollution; one is self-polishing coatings (SPCs), and the other is fouling release coatings (FRCs). FRCs do not prevent organisms from attaching, but the interfacial bond is weakened so that attached organisms are more easily removed by the hydrodynamic shear forces generated by the movement of the ship or by gentle ‘grooming’ devices. SPCs are the common protective measure for marine antifouling [8,15,16]. These coatings, typically hydrolyzed acrylic resins, gradually expose biocides on the surface as they erode, effectively killing fouling organisms [17,18,19]. However, their effectiveness is relatively short-lived. As the coating wears down, the underlying epoxy is gradually exposed to seawater. This is concerning because epoxy primers are highly biocompatible and vulnerable to biofouling. Therefore, protecting the epoxy undercoat is momentous once antifouling coatings fail. Recently, ultrasonic antifouling technology has increasingly gained attention and begun to show up in various marine settings [20,21,22]. Ultrasonic antifouling technology produces cavitation bubbles that generate high-temperature, high-pressure shock waves and microjets which impact the cellular structure and surface properties of microorganisms [23], including bacterial morphology [24,25], relative surface hydrophobicity [26,27,28], and Zeta potential [29]. Compared to traditional underwater mechanical scraping methods [30,31,32], ultrasonic antifouling technology offers the advantages of being proactive, non-toxic, environmentally friendly, and highly adaptable, which makes it suitable for various underwater structures such as ships, pipelines, and offshore platforms [20]. Despite its huge potential, there are limited studies on the impact of ultrasonic technology on the adhesion behavior of fouling microorganisms.

**Figure 1:**
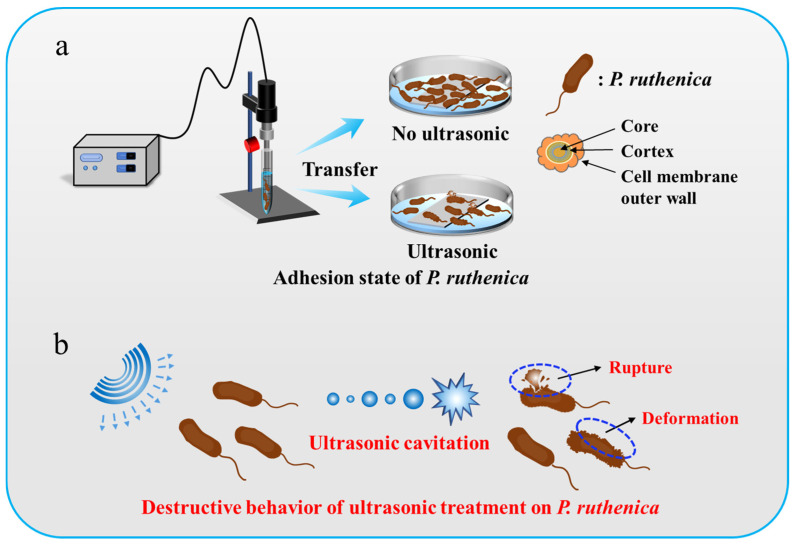
Schematic illustration of ultrasound-treated *Pseudoalteromonas*. (**a**) Adhesion of *Pseudoalteromonas* cells in absence and presence of ultrasonic treatment. (**b**) Destructive behavior of ultrasonic treatment on *Pseudoalteromonas* cells.

In this study, we investigated how ultrasonic technology affects the adhesion behavior of *Pseudoalteromonas* on a gel-like marine epoxy primer. Ultrasonic treatment for *Pseudoalteromonas* on a gel-like marine epoxy primer at a specific frequency and intensity produces numerous microbubbles due to ultrasonic cavitation which undergo formation, oscillation, growth, and collapse. Ultrasonic treatment generates instantaneous high temperature and pressure in a local area, which can burst the cell membrane and kill marine organisms, thereby diminishing the attachment of *Pseudomonas* to the gel-like marine epoxy primer (Figure 1). We systematically examined changes in the cell structure, particle size, Zeta potential, and surface wettability of *Pseudoalteromonas* before and after ultrasonic treatment at the optimal ultrasonic parameters based on response surface analysis. The results exhibited that ultrasonic treatment decreased the particle size of bacteria, weakened the Zeta potential of *Pseudoalteromonas*, and translated the surface wettability to hydrophobicity. As a whole, ultrasonic treatment significantly hindered the adhesion behavior of bacteria, offering a promising antifouling strategy for protecting hull surfaces. This study supports the adoption of ultrasonic technology in marine engineering, marine biology, and environmental protection, providing scientific evidence for sustainable marine environment management.

## 2. Results and Discussion

### 2.1. The Optimization of Ultrasonic Treatment Parameters

**Figure 2:**
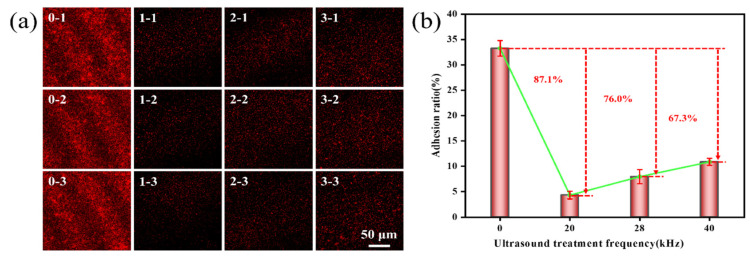
Effect of ultrasonic frequency on adhesion behavior of *Pseudoalteromonas*. (**a**) CLSM images of *Pseudoalteromonas* under different ultrasonic treatment frequencies: 0–1~0–3(0 kHz); 1–1~1–3 (20 kHz); 2–1~2–3 (28 kHz); 3–1~3–3 (40 kHz). (**b**) Curve of adhesion rate of *Pseudoalteromonas* with ultrasonic treatment frequency.

Ultrasonic frequency, power, and time will significantly affect the adhesion of *Pseudoalteromonas*. This study used Image J software win 64 to measure the mean fluorescence intensity of fluorescence images to assess the adhesion rate of *Pseudoalteromonas* on a gel-like marine epoxy primer under varying ultrasonic frequencies, powers, and time. The findings revealed that as the ultrasonic frequency decreased, the adhesion rate of *Pseudoalteromonas* on a gel-like marine epoxy primer also declined (Figure 2). In the control group (no ultrasonic treatment), the adhesion rate was 33.25 ± 1.53%. Treatment with 20 kHz ultrasound reduced the adhesion rate to 4.33 ± 0.75%. This resulted in an inhibition efficiency of 87.1%. Treatment with 28 kHz ultrasound resulted in an adhesion rate of 7.98 ± 1.40%, yielding an inhibition efficiency of 76.0%. Treatment with 40 kHz ultrasound led to an adhesion rate of 10.88 ± 0.69%, corresponding to an inhibition efficiency of 67.3%. These decreases in adhesion rates are attributed to the reduced adhesion and motility of *Pseudoalteromonas* following ultrasonic treatment [33,34], which hinders their adhesion to the gel-like marine epoxy primer. The highest inhibition efficiency was observed at 20 kHz ultrasound. As ultrasonic frequency increases, the interval between oscillations shortens, limiting the time available for cavitation bubbles to stretch and compress. It in turn reduces the volume change and energy storage in the cavitation bubbles, weakening the released energy against *Pseudoalteromonas* [35].

**Figure 3:**
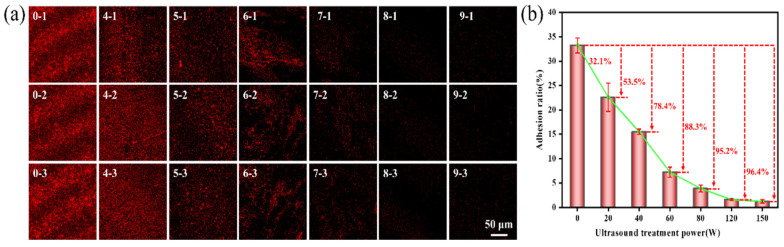
Effect of ultrasonic power on adhesion behavior of *Pseudoalteromonas*. (**a**) CLSM images of *Pseudoalteromonas* under different ultrasonic treatment powers: 0–1~0–3 (0 W); 4–1~4–3 (20 W); 5–1~5–3 (40 W); 6–1~6–3 (60 W); 7–1~7–3 (80 W); 8–1~8–3 (120 W); 9–1~9–3 (150 W). (**b**) Curve of adhesion rate of *Pseudoalteromonas* with ultrasonic treatment power.

As illustrated in Figure 3, the amount and rate of *Pseudoalteromonas* adhered to the gel-like marine epoxy primer decrease with a higher ultrasonic power. At an ultrasonic power of 80 W, the adhesion rate of *Pseudoalteromonas* was 3.89 ± 0.73%, yielding an inhibition efficiency of 88.3%. As ultrasonic power increased, inhibition efficiency approximately approached 100%. At 150 W, the adhesion rate decreased to 1.23 ± 0.37%, with an inhibition efficiency of 96.4%. Ultrasonic power influences the intensity and density of acoustic energy. As power increases, both the intensity and density of acoustic energy also rise. The higher acoustic energy intensity boosts gas supersaturation in the solution and increases the number of cavitation nuclei. Similarly, the increased energy density enhances energy storage within cavitation bubbles, amplifying the cavitation effect [36,37]. Therefore, increasing ultrasonic power progressively reduces the adhesion rate of *Pseudoalteromonas* on the gel-like marine epoxy primer.

**Figure 4:**
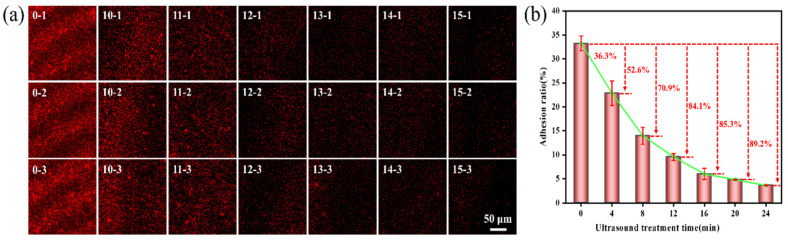
Effect of ultrasonic time on adhesion behavior of *Pseudoalteromonas*. (**a**) CLSM images of *Pseudoalteromonas* under different ultrasonic treatment times: 0–1~0–3 (0 min); 10–1~10–3 (4 min); 11–1~11–3 (8 min); 12–1~12–3 (12 min); 13–1~13–3 (16 min); 14–1~14–3 (20 min); 15–1~15–3 (24 min). (**b**) Curve of adhesion rate of *Pseudoalteromonas* with ultrasonic treatment time.

The impact of ultrasonic treatment time on the adhesion of *Pseudoalteromonas* on the gel-like marine epoxy primer is depicted in Figure 4, demonstrating that adhesion decreases with a longer treatment time. After 16 min of ultrasonic treatment, the adhesion rate was 6.02 ± 1.15%, resulting in an inhibition efficiency of 84.1%. Extending the treatment time to 24 min reduced the adhesion rate to 3.63 ± 0.08%, with an inhibition efficiency of 89.2%. As the ultrasonic treatment time increased, inhibition efficiency approached 90.0%. A longer treatment time allows for the accumulation of energy within cavitation bubbles before collapse and increases the formation and collapse of cavitation bubbles [38], which intensifies the cavitation effect.

According to the analysis of curves above, there is a significant decrease in the *Pseudoalteromonas* adhesion rate when ultrasonic frequency ranges from 20 kHz to 40 kHz, ultrasonic power ranges from 40 W to 80 W, and ultrasonic time ranges from 4 to 12 min. Design-Expert software 11 was employed to optimize ultrasonic treatment parameters, using the Box–Behnken design (BBD) method [39,40]. The factor levels for the response surface experiments are presented in Table 1.

Table 2 presents 17 different ultrasonic treatment schemes obtained through the BBD design. Data for the response variable (efficiency) were gathered experimentally. Polynomial regression was used to fit the relationship between experimental results and variables, leading to the following regression equation: $Y=59.17-8.41\times A+10.30\times B+15.90\times C+0.5907\times AB-0.0250\times BC+9.62\times A^{2}-3.35\times B^{2}-13.02\times C^{2}$, where Y represents the inhibition efficiency of ultrasonic treatment, A is the ultrasonic frequency, B is the ultrasonic power, and C is the ultrasonic time. By maximizing efficiency, the identified optimal process parameters were an ultrasonic frequency of 20 kHz, ultrasonic power of 75 W, and ultrasonic time of 10 min. These optimal parameters were then used to conduct further measurements of other surface properties of *Pseudoalteromonas*, as discussed in Section 3.3, Section 3.4 and Section 3.5.

An analysis of variance was performed on the three-factor, three-level experiment involving the ultrasonic treatment of *Pseudoalteromonas*. In Table 3, the interaction terms AB, AC, and BC refer to the interactions between ultrasonic frequency and power, frequency and time, and power and time, respectively. The F-values highlight the significance of each factor’s impact on the inhibition efficiency of ultrasonic treatment—the larger the F-value, the more significant the factor’s effect on inhibition efficiency. According to Table 3, the F-values for ultrasonic frequency, power, and time are 40.56, 59.73, and 142.34, respectively, indicating that the order of significance for the impact on inhibition efficiency is as follows: ultrasonic time > ultrasonic power > ultrasonic frequency. The *p*-values for frequency, power, and time are all less than 0.001, showing that each of the three factors significantly influences the inhibition efficiency of ultrasonic treatment. In contrast, the *p*-values for the interaction terms AB, AC, and BC are 0.7585, 0.4295, and 0.9897, respectively—all greater than 0.1. This suggests that the interaction terms do not significantly affect the inhibition efficiency of ultrasonic treatment. The *p*-value of the regression equation model is less than 0.001, indicating that the model is highly significant. The lack-of-fit *p*-value is 0.3243 (greater than 0.05), suggesting that the lack of fit is not significant [41]. Therefore, the regression equation model exhibits a good fit within the key regression region and demonstrates high reliability.

### 2.2. Effect of Ultrasonic Treatment on Pseudoalteromonas Morphology

**Figure 5:**
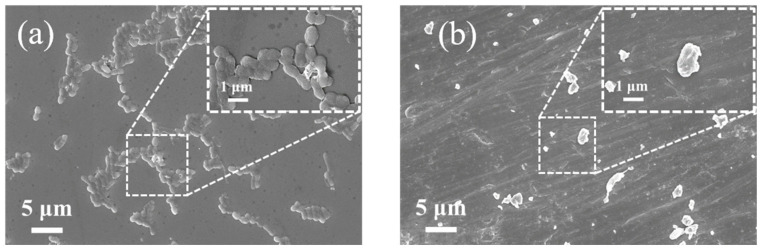
SEM images of *Pseudoalteromonas*. (**a**) SEM image of *Pseudoalteromonas* before ultrasonic treatment; (**b**) SEM image of *Pseudoalteromonas* after ultrasonic treatment.

The morphological structure of *Pseudoalteromonas* was closely examined using scanning electron microscopy (SEM). Figure 5a shows SEM images of *Pseudoalteromonas* before ultrasonic treatment. The image reveals that *Pseudoalteromonas* cells have an elliptical shape under normal growth conditions. They adhere, grow, and reproduce densely on the epoxy primer. Higher-magnification imaging (local image) shows smooth, intact outer cell walls covered with flagella, and cells secret extracellular polymeric substances (EPSs) on the substrate, allowing for reversible adhesion. After the optimized ultrasonic treatment, *Pseudoalteromonas* morphology was significantly damaged. Figure 5b shows that the outer cell wall became deformed and rough, and cells appeared fragmented, with numerous cell fragments visible. This damage is due to the cavitation effect of ultrasound. When the energy within the cavitation bubbles reaches a threshold, the bubbles collapse instantly, generating high temperatures up to 5500 K and pressures up to 50,000 kPa. Shock waves and microjets impact the bacterial surface, causing the deformation of the outer cell wall. Additionally, the collapse of cavitation bubbles is accompanied by high shear forces, triggering turbulence and fluid rotary motion in the solution. This further increases friction against the bacterial outer cell walls.

### 2.3. Ultrasound Effects on Pseudoalteromonas Relative Surface Hydrophobicity

To assess the impact of ultrasonic treatment on the relative surface hydrophobicity of *Pseudoalteromonas*, this study measured the optical density of *Pseudoalteromonas* suspensions using a UV-Vis spectrophotometer and calculated RSH using Formula (2). As Table 4 shows, untreated *Pseudoalteromonas* had an RSH of 7.2%, indicating strong hydrophilicity. After just one minute of ultrasonic treatment, the RSH increased rapidly to 26.3%, suggesting a gradual shift in some *Pseudoalteromonas* cells from hydrophilic to hydrophobic. With a longer treatment time, RSH slowly increased and stabilized. At a treatment time of 7 min, RSH stabilized at 35.4%, reflecting a significant rise in relative surface hydrophobicity compared to the untreated state. The lower RSH value in untreated *Pseudoalteromonas* results from the presence of numerous polar molecular groups (such as –OH) on the bacterial surface, which contribute to strong hydrophilicity. Ultrasonic treatment disrupts these polar groups. Additionally, water molecules were decomposed into ·H and ·OH radicals due to the high temperature and pressure from cavitation bubble collapse during treatment. The resulting ·OH radicals combine to form highly oxidative H_2_O_2_, causing the oxidation and inactivation of proteins and other organic substances. Consequently, the RSH of *Pseudoalteromonas* increases, making it less likely to adhere to the epoxy primer.

### 2.4. Ultrasound Effects on Pseudoalteromonas Particle Size and Zeta Potential

**Figure 6:**
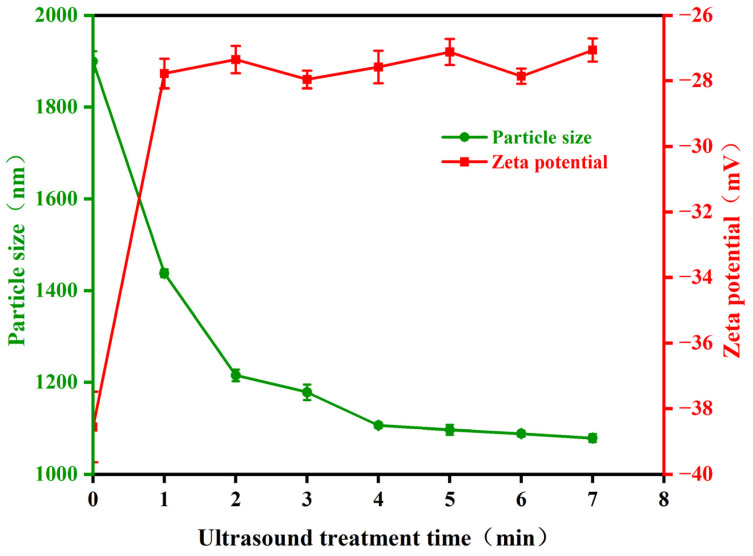
The change in *Pseudoalteromonas* particle size and Zeta potential with ultrasonic treatment time.

Bacterial cells contain proteins made of amino acids, which are zwitterions that can dissociate in solution into anions (carboxyl groups) and cations (amino groups). Typically, the pH of bacterial culture medium is higher than its isoelectric point, causing the bacteria to carry negative charge due to the disruption of charge balance on the surface. The changes in Zeta potential affect the stability of the sample system. To understand the impact of ultrasonic treatment on the particle size and Zeta potential of *Pseudoalteromonas*, this study measured these properties using a nanoparticle size analyzer. The results are presented in Figure 6. The average particle size of *Pseudoalteromonas* decreased with increasing ultrasonic treatment time. In the untreated condition, the average particle size was 1899.36 nm. After one minute of ultrasonic treatment, it rapidly decreased to 1437.67 nm. Following six times of one-minute ultrasonic treatment, the average particle size gradually stabilized at around 1080 nm. The absolute value of the Zeta potential of *Pseudoalteromonas* quickly decreased and stabilized as ultrasonic treatment time increased. Initially, the Zeta potential was −38.56 mV, a relatively high absolute value. After one to two minutes of treatment, it decreased rapidly to −27.35 mV. As treatment continued, the Zeta potential stabilized at around −27 mV. These results suggest that ultrasonic treatment damages the outer structural proteins of *Pseudoalteromonas*, reducing the absolute value of the Zeta potential. The reduced surface charge decreases the stability and dispersibility of the bacterial suspension, which hinders the adhesion, growth, and reproduction of *Pseudoalteromonas*.

All in all, the deformation of the outer cell wall, the increased RSH value, and the decreased average particle size and Zeta potential of *Pseudoalteromonas* under ultrasonic treatment were unfavorable for them to adhere to the epoxy primer. After ultrasonic treatment, the optimal adhesion inhibition rate reached 96.4%. These experimental results clearly indicate the application potential of ultrasonic technology in antifouling. Although our results were obtained at the laboratory scale, recent years have witnessed the engineering application of ultrasonic technology in sea pastures [42], offshore platforms [43], and so on. For hull surfaces, the installation of ultrasonic transducers with different channels can achieve the effective fouling prevention of organisms, as reported by Ji-Soo Park in 2018 [21]. Nevertheless, there are still challenges in the application of this technology. For example, the propagation of ultrasonic waves in the complex shape of marine structures is not uniform, and it is difficult to ensure that each part can receive sufficient intensity ultrasonic waves to prevent biological fouling. Moreover, the stability of ultrasonic equipment during operation is also a concern. After long-time continuous operation, electronic components inside the equipment may fail, resulting in unstable or interrupted ultrasonic output that will impair the antifouling effect.

## 3. Materials and Methods

### 3.1. Materials

*Pseudoalteromonas* was sourced from College of Ocean & Earth Sciences, Xiamen University, China. N-hexadecane, anhydrous ethanol, glutaraldehyde, phosphate-buffered saline (0.1 mol/L, pH of 7.0), and propidium iodide were bought from Aladdin Biochemical Technology Co., Ltd., (Shanghai, China). The 2216E liquid medium was bought from Haibo Biotechnology Co., Ltd., (Qingdao, China). The epoxy primer (N10) was obtained from Jotun Marine Coatings Co., Ltd., (Qingdao, China). The epoxy primer samples were made by mixing components A and B of N10 and brushing the mixture onto 20 mm × 20 mm glass slides. At the initial phase in the beginning of coating, it can be regarded as a pre-gel state, and as the gel process progresses, the component gradually binds tightly to the substrate.

### 3.2. Culture of Pseudoalteromonas

Dissolve 29.92 g of 2216E liquid culture medium in an Erlenmeyer flask with 800 mL of deionized water. Cover the flask with heat-resistant sealing film and sterilize it in an autoclave (DSX-30L-I, Shanghai ShenAn Medical instrument Factory, Shanghai, China) at 121 °C for 15 min. Once the medium cools to room temperature, it can be used as the nutrient solution for culturing *Pseudoalteromonas*. In a sterile environment, transfer approximately 40–45 mL of the 2216E liquid culture medium into a 50 mL centrifuge tube. Add 1 mL of the frozen bacterial suspension to the centrifuge tube. Incubate the mixture in a shaking incubator (MQD-M1G, Digi International, Shanghai, China) at 150 rpm and 30 °C for 24–72 h to produce the *Pseudoalteromonas* suspension.

### 3.3. Design of Ultrasonic Treatment Experiments

Ultrasonic treatment experiments on *Pseudoalteromonas* were carried out on the ultrasonic cell crusher purchased from Shanghai Micro Technology Co., Ltd., (Shanghai, China) which has a power range of 0–300 W. Three frequencies (20 kHz, 28 kHz, and 40 kHz) are available. These experiments were conducted according to the factor levels listed in Table 5. Each experiment had three parallel samples. The effects of ultrasonic frequency, power, and time on the adhesion rate of *Pseudoalteromonas* were investigated. To determine the bacterial concentration, the absorbance of the *Pseudoalteromonas* suspension was measured using a UV-Vis spectrophotometer (UV-2600i, Shimadzu, Beijing, China) at a wavelength of 600 nm, yielding an optical density value of 0.510, which corresponds to a bacterial concentration of 10^6^ cfu/mL. The epoxy-coated samples were placed in a 6-well plate and sterilized with UV light. A total of 10 mL of *Pseudoalteromonas* suspension was then subjected to ultrasonic treatment in each of the single-factor experiments, with the control group remaining untreated. The treated suspensions were added to the 6-well plate and incubated at 30 °C in a biochemical incubator (SHP-150, SMA Solar Technology, Shanghai, China) for 72 h. After incubation, the samples were removed and washed three times with deionized water to eliminate unattached *Pseudoalteromonas*. The bacteria were then fixed with a 2.5% glutaraldehyde solution for 4 h at 4 °C. The samples were rinsed twice with deionized water to remove any residual glutaraldehyde solution. Next, the *Pseudoalteromonas* were stained with a 50 μg/mL propidium iodide (PI) solution for 15 min and then washed again with deionized water to remove any residual PI. The treated samples were observed under a confocal laser scanning microscope (CLSM, Leica TCS SP8, Wetzlar, Germany) for fluorescence imaging. Fluorescence images were captured from various areas of the samples and processed using ImageJ software win 64 to calculate the adhesion rate of *Pseudoalteromonas*. The inhibition efficiency (E) of the ultrasonic treatment was calculated according to Formula (1).

(1) $$E=(1-\frac{E_{b}}{E_{a}})\times 100\%$$

where E_a_ represents the adhesion rate of *Pseudoalteromonas* on epoxy primer without ultrasonic treatment, and E_b_ represents the adhesion rate after ultrasonic treatment.

Based on the single-factor experiments described in Table 5, the range of level values for each ultrasonic parameter was established. The Box–Behnken design in Design-Expert software 11 was used to optimize the ultrasonic treatment parameters. The inhibition efficiency (E) was the response variable (Y), while ultrasonic frequency (A), ultrasonic power (B), and ultrasonic time (C) were independent variables. A three-factor quadratic equation modeled the relationship between inhibition efficiency and ultrasonic parameters, followed by range analysis. The regression equation was numerically optimized using the “Optimization” function in Design-Expert. Efficiency was set as the parameter to optimize, with the “Goal” set to maximize efficiency. This approach was used to determine the optimal ultrasonic treatment process parameters.

### 3.4. Microstructure of Pseudoalteromonas

The microstructure of *Pseudoalteromonas* was observed and photographed using a scanning electron microscope (Zeiss, Sigma 500, Jena, Germany) at 8 kV, with magnifications ranging from 100× to 4000×. For SEM imaging, *Pseudoalteromonas* samples were prepared as follows: the ultrasonic-treated suspension was centrifuged at 5000× *g* rpm and 4 °C for 10 min using a high-speed refrigerated tabletop centrifuge (H1750R, CENCE, Hunan, China) to obtain a cell pellet. The pellet was washed twice with sterile deionized water and then fixed overnight at 4 °C with a 2.5 wt% glutaraldehyde solution. After fixation, samples were washed three times with phosphate-buffered saline solution (pH of 7.0) and dehydrated using a series of gradient ethanol solutions (25 wt%, 50 wt%, 75 wt%, 90 wt%, 100 wt%) for 5 min each. The samples were then dried in a blast drying oven (GFX-9073A, Moderner, Shanghai, China) at 35 °C for 12 h. Finally, the prepared samples were observed and imaged using the scanning electron microscope after spray-gold treatment.

### 3.5. Measurement of Relative Surface Hydrophobicity (RSH) of Pseudoalteromonas

The relative surface hydrophobicity of *Pseudoalteromonas* was measured using n-hexadecane. First, using 2216E liquid medium as a reference, the initial optical density (OD) of the untreated *Pseudoalteromonas* suspension was measured at a wavelength of 600 nm using a UV-Vis spectrophotometer (UV-2600i, Shimadzu, Beijing, China), yielding a value of A_0_. Next, 3 mL of ultrasonic-treated *Pseudoalteromonas* suspension was mixed with 0.6 mL of n-hexadecane. The mixture was vortexed for 30 s and allowed to stand for 15 min. Then, the OD of the aqueous phase of the mixture was measured again at a wavelength of 600 nm, yielding a value of A_f_. The change in OD reflects the shift in the relative surface hydrophobicity of *Pseudoalteromonas*. Relative surface hydrophobicity is defined as the proportion of hydrophobic cells to the total number of cells. It can be calculated using Formula (2) to determine how relative surface hydrophobicity changes after ultrasonic treatment.

(2) $$RSH\left(\%\right)=(1-\frac{A_{f}}{A_{0}})\times 100\%$$

### 3.6. Measurement of Particle Size and Zeta Potential of Pseudoalteromonas

The changes in the particle size and Zeta potential of *Pseudoalteromonas* in aqueous solution (pH of 7.0) before and after ultrasonic treatment were measured by nanoparticle size and Zeta potential analyzer (NanoBrook 90Plus PALS, Brookhaven Instruments, Holtsville, NY, USA). The specific method is as follows: firstly, *Pseudoalteromonas* was diluted with deionized water to an absorbance value of about 0.3; then, 1.8–3.0 mL suspensions were placed in a cuvette, and the particle size and Zeta potential value were measured at room temperature. During the test, the arithmetic average value after 10–20 automatic tests by the software was used as the final measurement result. The specific method is as follows: DLS particle sizing measurement was selected. The parameters were set as follows: the wavelength was 659.0 nm, the temperature was 25.0 °C, the duration was 120 s, and the equilibration time was 300 s. The starting point temperature was set to 25.0 °C, the end temperature was 35.0 °C, and the temperature increment was 2.0 °C. The selected solvent (liquid) was water.

## 4. Conclusions

This study investigated the adhesion behavior of *Pseudoalteromonas* on a gel-like marine epoxy primer under ultrasonic treatment. According to the experimental results above, some instructive conclusions could be obtained. The optimized parameters were 20 kHz frequency, 75 W power, and 10 min treatment time. After seven minutes of treatment, *Pseudoalteromonas* experienced the deformation and partial rupture of the cell membrane, leading to cellular damage. The particle size of the bacteria decreased rapidly, and relative surface hydrophobicity changed from 7.2% to 35.4%, which proved the reduced adhesion capability of the bacteria on hydrophilic surfaces. After one minute of ultrasonic treatment, the Zeta potential of *Pseudoalteromonas* weakened significantly which likely resulted from the disruption of ultrasonic treatment on the structural proteins of the cell membrane, lowering the bacteria’s surface charge. Thus, the stability and dispersion of the bacterial suspension were destructed, thereby hindering *Pseudoalteromonas* adhesion, growth, and reproduction. This study demonstrated that ultrasonic treatment can inhibit the development of microbial biofilm and reduce biofouling formation by altering the adhesion ability of bacteria on material surfaces. With its efficiency, non-toxicity, and adaptability to different biological environments, ultrasonic treatment offers valuable theoretical insights and practical guidance for marine antifouling applications.

## Figures and Tables

**Table 1:** Factor level table of response surface test for ultrasonic treatment of *Pseudoalteromonas*.

Level	Frequency/kHz	Power/W	Time/min
−1	20	40	4
0	28	60	8
1	40	80	12

**Table 2:** Results of three-factor and three-level experiment on *Pseudoalteromonas* with ultrasonic treatment.

Trial	Frequency/kHz	Power/W	Time/min	Efficiency/%
1	20	40	8	64.2
2	20	80	8	87.1
3	20	60	4	46.3
4	20	60	12	78.5
5	28	80	4	36.3
6	28	40	4	18.8
7	28	60	8	57.4
8	28	60	8	61.2
9	28	40	12	53.5
10	28	80	12	70.9
11	28	60	8	63.3
12	28	60	8	65.6
13	28	60	8	58.7
14	40	40	8	43.2
15	40	60	12	62.6
16	40	80	8	67.3
17	40	60	4	35.7

**Table 3:** Three-factor, three-level test range analysis table of ultrasonic treatment for *Pseudoalteromonas*.

Factor	Sum of Square	Freedom	Mean Square	F-Value	*p*-Value	Significance Level
Model	4446.06	9	494.01	35.47	<0.0001	Significant
A	566.16	1	566.16	40.65	0.0004	
B	831.84	1	831.84	59.73	<0.0001	
C	1982.36	1	1982.36	142.34	<0.0001	
AB	1.42	1	1.42	0.1022	0.7585	
AC	9.79	1	9.79	0.7029	0.4295	
BC	0.0025	1	0.0025	0.0002	0.9897	
A^2^	351.88	1	351.88	25.27	0.0015	
B^2^	47.11	1	47.11	3.38	0.1085	
C^2^	713.77	1	713.77	51.25	0.0002	
Residual	97.49	7	13.93			
Lack of fit	53.04	3	17.68	1.59	0.3243	Not significant

**Table 4:** Changes in relative surface hydrophobicity of *Pseudoalteromonas* before and after ultrasonic treatment.

Time/min	0	1	2	3	4	5	6	7
A_0_	0.535	0.532	0.534	0.533	0.535	0.534	0.534	0.531
A_f_	0.496	0.392	0.376	0.371	0.359	0.347	0.343	0.343
RSH (%)	7.2	26.3	29.5	30.4	32.9	35.0	35.8	35.4

**Table 5:** Single-factor experiment design table of ultrasonic treatment for *Pseudoalteromonas*.

Number	Frequency/kHz	Power/W	Time/min
1	20	80	8
2	28	80	8
3	40	80	8
4	28	20	12
5	28	40	12
6	28	60	12
7	28	80	12
8	28	120	12
9	28	150	12
10	28	80	4
11	28	80	8
12	28	80	12
13	28	80	16
14	28	80	20
15	28	80	24

## Data Availability

The data presented in this study are available in the article itself.
